# Involvement of eNAMPT/TLR4 inflammatory signaling in progression of non-alcoholic fatty liver disease, steatohepatitis, and fibrosis

**DOI:** 10.1096/fj.202201972RR

**Published:** 2023-03

**Authors:** Belinda L. Sun, Xiaoguang Sun, Carrie L. Kempf, Jin H. Song, Nancy G. Casanova, Sara M. Camp, Vivian Reyes Hernon, Michael Fallon, Christian Bime, Diego R. Martin, Cristina Travelli, Donna D. Zhang, Joe G. N. Garcia

**Affiliations:** 1 Department of Pathology, College of Medicine, University of Arizona, Tucson, Arizona, USA; 2 Department of Medicine, College of Medicine, University of Arizona, Tucson, Arizona, USA; 3 Department of Medicine, College of Medicine, University of Arizona, Phoenix, Arizona, USA; 4 Department of Radiology and the Translational Imaging Center, Houston Methodist Hospital and the Houston Methodist Research Institute, Houston, Texas, USA; 5 Department of Drug Science, University of Pavia, Pavia, Italy; 6 Department of Pharmacology and Toxicology, College of Pharmacy, University of Arizona, Tucson, Arizona, USA

**Keywords:** damage-associated molecular pattern protein, extracellular nicotinamide phosphoribosyltransferase, non-alcoholic fatty liver disease, non-alcoholic steatohepatitis, toll-like receptor 4

## Abstract

Although the progression of non-alcoholic fatty liver disease (NAFLD) from steatosis to steatohepatitis (NASH) and cirrhosis remains poorly understood, a critical role for dysregulated innate immunity has emerged. We examined the utility of ALT-100, a monoclonal antibody (mAb), in reducing NAFLD severity and progression to NASH/hepatic fibrosis. ALT-100 neutralizes eNAMPT (extracellular nicotinamide phosphoribosyltransferase), a novel damage-associated molecular pattern protein (DAMP) and Toll-like receptor 4 (TLR4) ligand. Histologic and biochemical markers were measured in liver tissues and plasma from human NAFLD subjects and NAFLD mice (streptozotocin/high-fat diet—STZ/HFD, 12 weeks). Human NAFLD subjects (*n* = 5) exhibited significantly increased NAMPT hepatic expression and significantly elevated plasma levels of eNAMPT, IL-6, Ang-2, and IL-1RA compared to healthy controls, with IL-6 and Ang-2 levels significantly increased in NASH non-survivors. Untreated STZ/HFD-exposed mice displayed significant increases in NAFLD activity scores, liver triglycerides, NAMPT hepatic expression, plasma cytokine levels (eNAMPT, IL-6, and TNFα), and histologic evidence of hepatocyte ballooning and hepatic fibrosis. Mice receiving the eNAMPT-neutralizing ALT-100 mAb (0.4 mg/kg/week, IP, weeks 9 to 12) exhibited marked attenuation of each index of NASH progression/severity. Thus, activation of the eNAMPT/TLR4 inflammatory pathway contributes to NAFLD severity and NASH/hepatic fibrosis. ALT-100 is potentially an effective therapeutic approach to address this unmet NAFLD need.

## 1 | INTRODUCTION

Caloric excess and sedentary lifestyles have led to a global epidemic of obesity, metabolic syndrome, and non-alcoholic fatty liver disease (NAFLD). NAFLD is a major public health issue^1,2^ in the United States and globally affects up to 25% of the world's adults^3,4^ and is associated with increased liver-related morbidity and mortality, and increased risk of cardiovascular disease, hepatic cancers, type 2 diabetes mellitus, and chronic kidney disease.^1,5^ Approximately 40% of patients with NAFLD progress from simple fatty liver (steatosis) to non-alcoholic steatohepatitis (NASH), and subsequently to hepatic fibrosis/cirrhosis with increased risk of hepatocellular carcinoma (HCC).^6,7^ The complex factors influencing NAFLD development and subsequent progression remain poorly understood but are multifactorial involving genetic variations, lipid peroxidation, oxidative stress, and imbalances in gut microbiota. Importantly, a critical role in dysregulated innate immune responses is now widely recognized.^8,9^ Although the therapeutic pipeline for NAFLD/NASH is expanding,^10,11^ this remains an unmet need as there are no FDA-approved medications for NAFLD.

Utilizing systems biology and genomic strategies, we previously identified extracellular nicotinamide phosphoribosyltransferase (eNAMPT) as a novel damage-associated molecular pattern protein (DAMP)^12^ that regulates innate immunity-mediated inflammation and pro-fibrosis pathways.^12–14^ We demonstrated that eNAMPT ligates the pathogen recognition receptor, Toll-like receptor 4 (TLR4), to induce NFkB-mediated inflammatory pathways^12,15^ that are involved in multiple inflammatory pathobiologies including ARDS,^14,16–20^ radiation,^13,21^ pulmonary hypertension,^22–24^ systemic lupus vasculitis,^25^ and cancers such as lung, breast, leukemia,^26,27^ and prostate cancer.^28,29^ In preclinical inflammatory/fibrosis models, a humanized eNAMPT-neutralizing mAb, ALT-100, dramatically reduces organ injury and fibrosis.^13,14,16,18,21,24,25^

eNAMPT is prominently released from multiple cells including innate immunity effector cells, epithelial cells, and endothelial cells^18^ as well as adipocytes as the adipokine, visfatin, with eNAMPT plasma levels increased in patients with obesity, metabolic syndrome insulin resistance, and type 2 diabetes mellitus.^18,30–32^ The current study was designed to investigate eNAMPT involvement in driving NAFLD severity and progression to hepatic fibrosis. Our results indicate significant increases in liver and blood eNAMPT expression in both human NAFLD subjects and in a streptozotocin (STZ)- and diet-driven murine NASH model. STZ/HFD mice receiving the eNAMPT-neutralizing ALT-100 mAb (weeks 9–12) exhibited significant decreases in NAFLD activity scores, liver triglycerides, plasma eNAMPT, IL-6, and TNFα levels, with reduced hepatic injury (hepatocyte ballooning) and fibrosis (Sirius staining) compared to untreated STZ/HFD mice. These studies indicate the eNAMPT/TLR4 inflammatory pathway activation as a key contributor to NAFLD development and progression to hepatic fibrosis. The DAMP-neutralizing ALT-100 mAb appears to be an effective therapeutic approach to address the unmet need for novel NAFLD treatments.

## 2 | METHODS

### 2.1 | Reagents and antibodies

Reagents were obtained from Sigma-Aldrich (St. Louis, MO) unless specifically stated. Details of the eNAMPT mAb (ALT-100) provided by Aqualung Therapeutics (Tucson, AZ) were previously reported.^16,18,28^

### 2.2 | Human subjects with NAFLD

Human tissues and plasma samples were obtained from the Tissue Acquisition and Cellular/Molecular Analysis Shared Resources Repository at the University of Arizona Department of Pathology. All methods were performed in accordance with the relevant guidelines and regulations of the University of Arizona College of Medicine Institutional Review Board and Banner-University Medical Center (IRB #1712117680). The study only used repository tissues without interaction with patients and without intervention to patients' management, thus consent was waived (informed consent waiver was approved by the University of Arizona College of Medicine and Banner-University Medical Center Institutional Review Board (IRB #1712117680)). Patient information was anonymized and de-identified prior to analysis.

Archived paraffin blocks of liver biopsy tissues were retrospectively collected from human subjects with NAFLD (*n* = 5) who exhibited pathologically and clinically diagnosed NAFLD (January to June 2013) and clinically diagnosed metabolic syndrome with clinical characteristics as reported in Table 1. Liver specimens collected for donor liver transplant from age-matched subjects (*n* = 5) without medical record evidence of diabetes, obesity, metabolic syndrome, or chronic liver disease were utilized for comparison.

Plasma samples were collected from a second NAFLD cohort (*n* = 67, Table 2) with clinical-diagnosed NASH and from healthy controls (*n* = 78, Table 2). Thirty-five of the 67 NASH subjects also had Type 2 diabetes as a comorbidity.

### 2.3 | Streptozotocin/high-fat diet (STZ/HFD) murine NASH model

All methods and experiments were performed in accordance with the relevant guidelines and regulations of the University of Arizona Institutional Animal Care and Use Committees, in addition to relevant ARRIVE guidelines.

C57Bl/6 male mice (Jackson Lab, California, USA) (*n* = 8 mice/group) received a single subQ injection of 200 μg of streptozotocin (STZ, Sigma, USA) on postnatal day 2 followed by high fat (HFD; 60% wt/wt fat; 58Y1; Test Diets, St. Louis, MO) ad libitum beginning at postnatal 4 weeks (day 28 ± 2). Prior studies utilizing this model showed STZ/HFD mice to exhibit relatively stable body weights with gradual liver weight increases, consistent with fatty liver changes and the accumulation of triglyceride.^33,34^ Elevated serum glucose and serum liver enzymes, alanine aminotransferase (ALT), and aspartate transaminase (AST) are observed in STZ/HFD mice at 12 weeks consistent with acute hepatitis and hepatocyte injury, findings that confirm a murine NAFLD model that successfully recapitulates human steatohepatitis and NAFLD.^35^

All STZ/HFD-exposed NASH mice were randomized into three groups (8 mice/group) based on body weights at 9 weeks of age (day 63 ± 2). An additional group of unexposed mice (*n* = 5) served as controls. Beginning in week 9, STZ/HFD-exposed mice received weekly intraperitoneal (IP) injections of either vehicle (human IgG, 0.4 mg/kg, *n* = 8) or the humanized eNAMPT-neutralizing mAb, ALT-100 (0.4 mg/kg, *n* = 8), from week 9 to week 12, when all mice were sacrificed. Throughout the entire 12-week experimental period, mice were monitored closely, weighed twice a week, and maintained on a 12 h light/12 h dark cycle. On achieving the study endpoints at the beginning of week 12, mice were anesthetized and euthanized in a manner consistent with the American Veterinary Medical Association Guidelines on Humane Euthanasia by using ketamine (100 mg/kg) and xylazine (5 mg/kg) anesthesia followed by exsanguination. Livers were collected for H&E staining and pathologic examination for NAFLD activity score, NAMPT immunohistochemistry, Sirius Red staining for areas of fibrosis, and liver triglyceride measurements (E-test kit, Fujifilm Wako, Japan). Blood samples were collected for ELISA analysis for eNAMPT, IL-6, and TNFα measurements, and liver function tests (alanine transaminase or ALT). Animals showing >25% body weight loss within a week or >20% body weight loss compared to previous day were euthanized ahead of study termination. Mice in all groups were sacrificed at 12 weeks of age. The NAFLD activity score is calculated by grading steatosis (0–3), hepatocyte ballooning (0–2), and lobular inflammation (0–3).

### 2.4 | Hepatic immunohistochemistry and semi-quantitative analysis

Paraffin-embedded blocks of human and murine liver tissues were processed (4 μm paraffin tissue sections) with six sequential tissue sections per tissue block. Hepatic tissue sections were stained for collagens using Picro Sirius Red Stain Kit (Abca, Cambridge, MA) following manufacturer's instructions. Paraffin sections for immunohistochemistry were sequentially incubated with a rabbit anti-human NAMPT polyclonal antibody with a dilution of 1:1000 (Bethyl Laboratories, Inc, Cat #A300-A375A, Montgomery, TX), HRP-conjugated ABC kit (VECTASTAIN ABC HRP kit, VECTOR Laboratories, Burlingame, CA), followed by use of DAB as detection reagent (VECTASTAIN DAB kit, VECTOR Laboratories, Burlingame, CA). Stained slides from each tissue block were counterstained with hematoxylin for morphologic examination, and one parallel set of stained slides was processed without counterstaining for NAMPT immunoreactivity (repeated a minimum of three times) using Image J as previously reported.^28^ H&E-stained slides, NAMPT-immunostained with counterstain slides, and NAMPT-immunostained without counterstain slides were reviewed by a pathologist specializing in hepatic disease.

The intensity of NAMPT immunostaining or Sirius Red staining in liver tissue sections was determined using ImageJ Fiji software (version 1.2; WS Rasband, NIH, Bethesda, MD). The images were captured using a binocular Leica light microscope (Leica^™^ DM2500) using a bright-field and CCD color video camera (Leica DFC320) attached to a computer system and uploaded to ImageJ software. At least six random areas of hepatocyte parenchyma were selected using the “Selecting” tool in Image J software. Following the standard recommended protocol,^36,37^ the mean gray value at each selected area was measured^37^ using the “Measurement” tool in ImageJ to represent NAMPT immunostaining intensity. The acellular areas of each slide were selected and measured as background gray value which was used to normalize the NAMPT-immunostaining intensity. The gray values of staining were quantified and presented as mean Gray Value ± SEM. Data were compared between NAFLD and control livers.

### 2.5 | Biochemical analyses and immunoblotting

Proteins within hepatic tissue lysates were separated by SDS-PAGE, transferred to PVDF membranes (100 V for 1.5 h), and immunoreacted with a rabbit anti-human NAMPT polyclonal antibody (1:10 000, Bethyl Laboratories, Inc., Cat #A300-A375A, Montgomery, TX), SNAI1 (Cell Signaling Technology; cat # 3879S), TGFβ (Cell Signaling Technology; cat # 3711S), p-SMAD1/5/9 (Cell Signaling Technology; cat # 13820S), NRF2 (Invitrogen; cat # PA5-27882), and anti-mouse GAPDH or β-actin (Millipore Sigma; cat# A3854) antibody as described previously.^15^ Immunoreactive proteins were detected with the enhanced chemiluminescent detection system according to the manufacturer's directions (Amersham, Little Chalfont, UK) and quantified using NIH ImageJ analysis software.

### 2.6 | Biomarker detection in blood

Human plasma levels of eNAMPT, IL-6, angiopoietin-2 (ANG2), and IL-1RA were measured using a mesoscale ELISA platform (Meso Scale Diagnostics, U-PLEX Personalized Multiplexing, Rockville, Maryland, USA) as previously reported.^38–40^ The U-PLEX platform was utilized for plasma measurements of murine biomarkers eNAMPT, IL-6, KC (i.e., IL-8), and TNFα as previously reported.^13,18,21^ Each biotinylated antibody (10 μg/mL, specific for each analyte) was mixed with a different linker for each analyte and incubated for 30 min at 25°C. The reaction was terminated with 200 μL of free biotin solution and 600 μL of the 10× U-PLEX linked biotinylated antibody solution, with 50 μL of coating solution added to each well in 96-well plate and incubated for 1 h (800 rpm shaking, 25°C). After washing, each well was supplemented with 25 μL of diluent and 25 μL of calibrator or samples/standards, and incubated for 1 h (800 rpm shaking, 25°C). After washing (TBS-T), each well was supplemented with 50 μL/well of 1× detection antibody solution, incubated for 1 h, washed, and supplemented with 2× Read Buffer T followed by plate imaging and calculation of the absolute concentration values based on standards.

### 2.7 | NASH severity scores

NASH severity was determined by NAFLD activity score (NAS) and hepatic fibrosis staging. NAS components include (i) steatosis severity scored as: (1)—mild, 5%–33%; (2)—moderate, 33%–66%; and (3)—severe, >66%; (ii) lobular inflammation severity scored as: (1)—<2 foci/200×, (2)—2–4 foci/200×, and (3)—>4 foci/200×; and (iii) hepatocyte ballooning severity scored as: (1)—few ballooning cells and (2)—prominent ballooning of cells. Hepatic fibrosis staging was scored as (1) with perisinusoidal or periportal fibrosis, including 1A mild perisinusoidal, 1B moderate perisinusoidal, and 1C portal/periportal fibrosis; (2) with perisinusoidal and periportal fibrosis; (3) with bridging fibrosis; and (4) with cirrhosis.^41^

### 2.8 | Statistical analysis

Statistical tests were performed using Bonferroni multiple-comparison test. *p* values <.05 were considered statistically significant. The student *t*-tests and ANOVA were performed using GraphPad Prism version 6.0 for the comparisons of NAMPT immunostaining intensity, Sirius Red staining intensity, triglyceride levels, NAFLD activity score among NAFLD, and control groups. The Mann–Whitney *U* test for independent samples or non-parametric test was used for the comparison of human plasma levels (eNAMPT, IL-6, ANG2, and IL-1RA) and NAFLD mice values (eNAMPT, IL-6, KC, and TNFα). A *p*-value <.05 was statistically significant.

## 3 | RESULTS

### 3.1 | Elevated NAMPT expression in hepatic tissues and blood in NAFLD subjects

**FIGURE 1:**
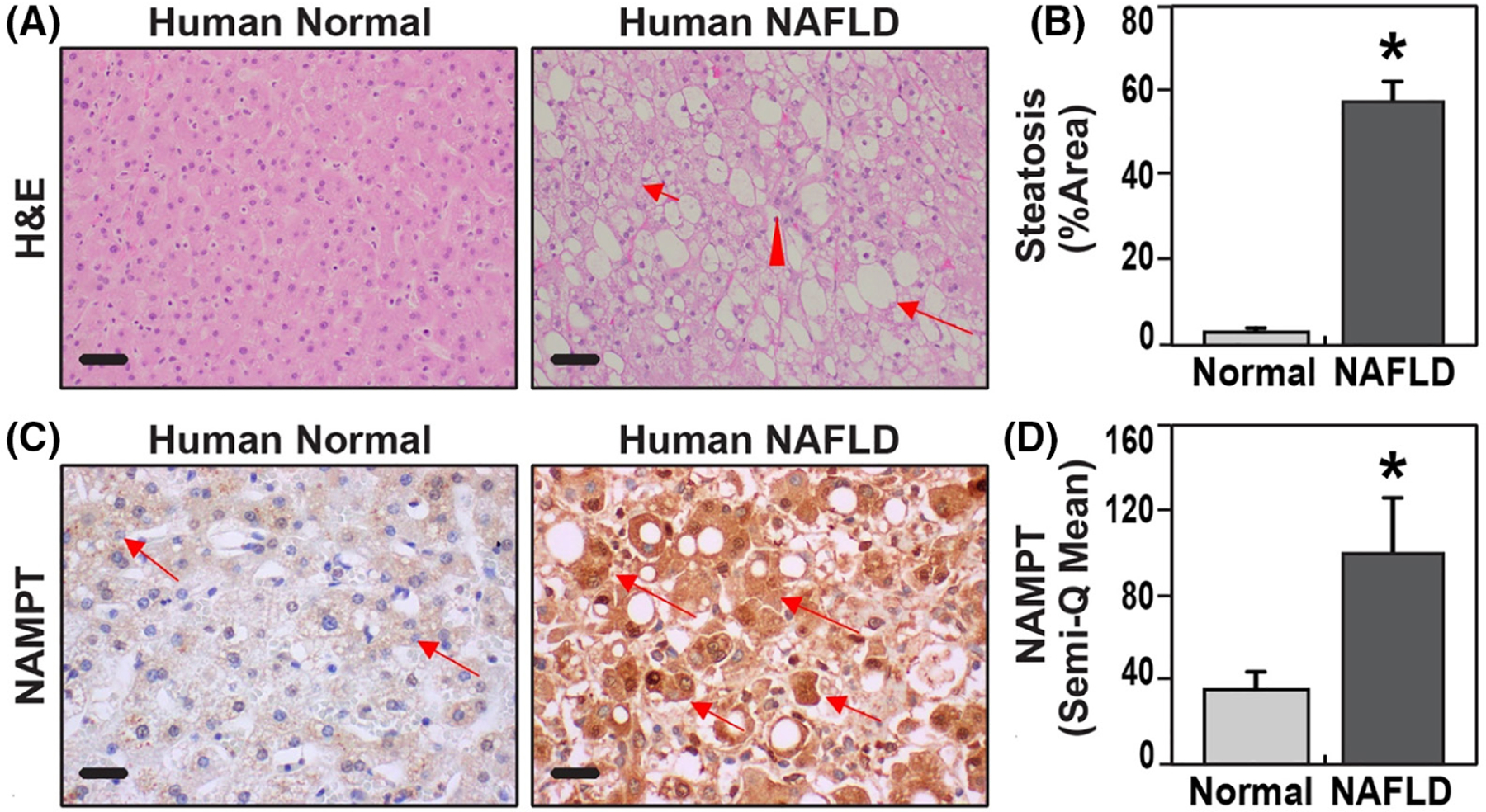
Elevated NAMPT hepatic expression in human subjects with NAFLD. (A) Normal liver tissue showing hepatocytes without steatosis (Human Normal). NAFLD liver tissue showing macrovesicular steatosis (long arrow), hepatocyte ballooning (short arrow), and focal lobular inflammation (arrowhead) (Human NAFLD) (bar—50 μm). (B) Semi-quantification of steatosis in hepatocytes showing significantly higher percentages of hepatocytes involved with lipid accumulation in NAFLD livers compared with normal livers. (C) Immunohistochemistry (IHC) for NAMPT showed mild positivity (arrow) in normal liver tissue (Human Normal), but strong positivity (arrows) in liver with NAFLD (Human NAFLD). (D) Image J software semi-quantitation of NAMPT immunostaining intensity represented as mean gray value confirmed significantly higher NAMPT staining in human NAFLD liver tissues compared to normal liver tissues (*p* < .05, *n* = 5) (bar—20 μm).

Evaluation of human NAFLD liver tissues by H&E staining (*n* = 5, Table 1) showed abnormal hepatic lobular structure, macrovesicular steatosis, hepatocyte ballooning, spotty lobular hepatitis, and pericellular fibrosis when compared to control human liver tissues (Figure 1A,B). Moderate-to-severe steatosis involving 40% to 70% of hepatocytes was identified in NAFLD liver tissues (Figure 1A,B). Immunohistochemical (IHC) analysis detected minimal NAMPT expression in control human liver tissues (Figure 1C,D). In contrast, NAMPT expression in human NAFLD livers was markedly elevated in hepatocytes and in infiltrating inflammatory cells (Figure 1C,D). Quantitation of NAMPT immunostaining intensity indicated a significantly greater NAMPT expression in NAFLD liver tissues compared to morphologically normal control liver tissues (Figure 1D).

**FIGURE 2:**
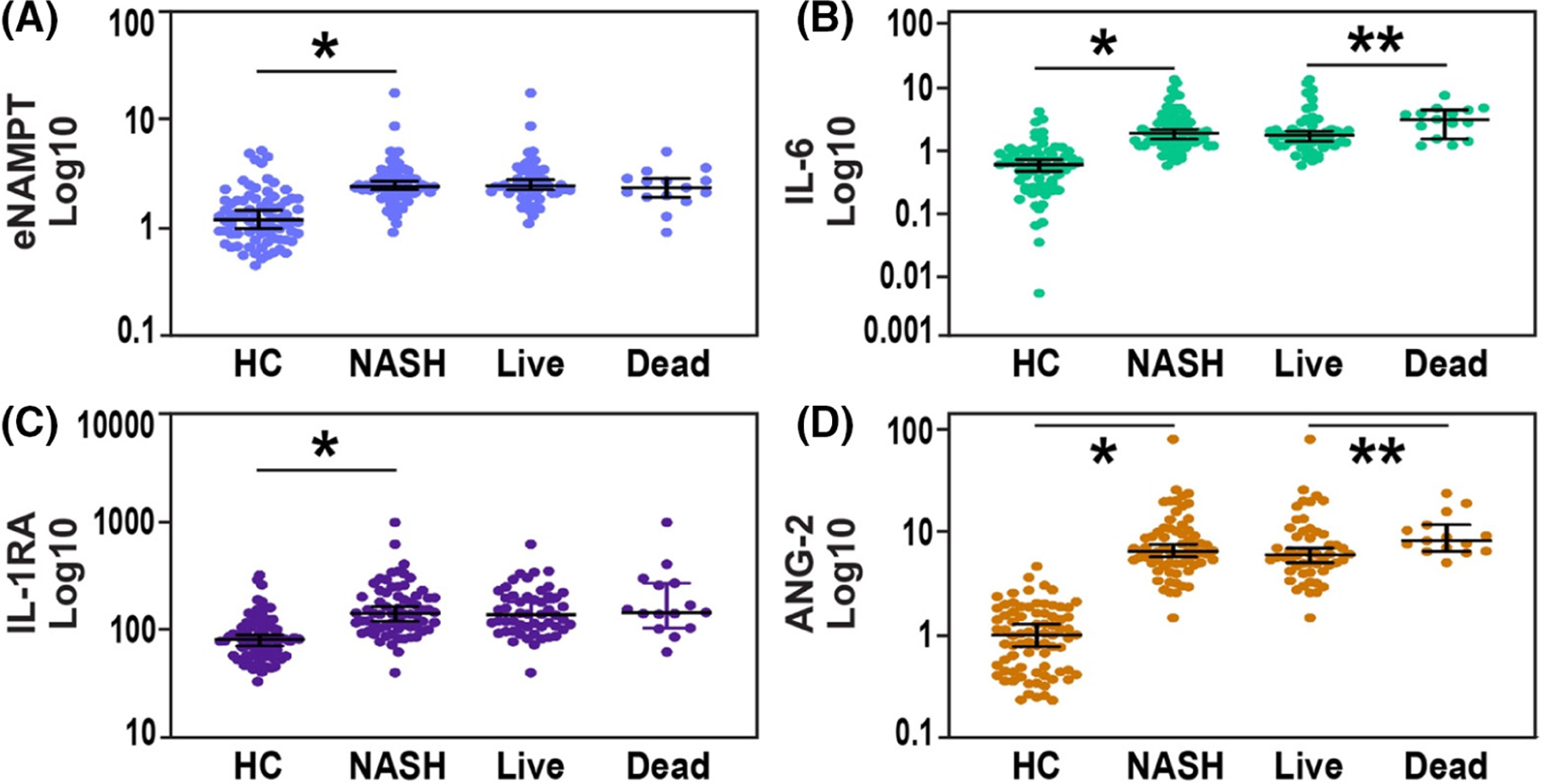
Elevated plasma NAMPT and cytokine in human subjects with NAFLD. (A) Comparison of plasma eNAMPT levels in NAFLD patients (*n* = 67) and healthy controls (*n* = 78) showed significantly higher eNAMPT in NAFLD patients (*p* < .05). NAFLD subjects were grouped into surviving subjects and non-survivors and showed both groups have significantly higher levels of eNAMPT compared to healthy controls, although there were no significant differences between survivors and non-survivors. (B) Comparison of plasma IL-6 levels in NAFLD patients, surviving NAFLD subjects, non-surviving NAFLD subjects, and healthy controls (*p* < .05). (C) Comparison of plasma IL-1RA levels in NAFLD patients, surviving NAFLD subjects, non-surviving NAFLD subjects, and healthy controls (*p* < .05). (D) Comparison of plasma ANG2 levels in NAFLD patients, surviving NAFLD subjects, non-surviving NAFLD subjects, and healthy controls (*p* < .05).

Consistent with these results, median eNAMPT plasma levels were significantly elevated in human NASH subjects (*n* = 67, 2.40 ng/mL) compared to healthy controls (*n* = 78, 1.20 ng/mL) (Figure 2A). Consistent with the inflammatory nature of NASH pathobiology, markedly elevated plasma levels of IL-6, IL-1RA, and Ang-2 were detected in NASH subjects (Figure 2B–D) compared to healthy controls (each *p* < .05). In addition, significant elevations in IL-6 and Ang-2 levels were noted in NASH non-survivors (*n* = 52) compared to survivors (*n* = 15) (Figure 2B,D). Plasma eNAMPT and IL-1RA levels were not significantly different between NASH survivors and non-survivors (Figure 2A,C).

### 3.2 | Elevated NAMPT expression in hepatic tissues and blood in preclinical murine NAFLD models

**FIGURE 3:**
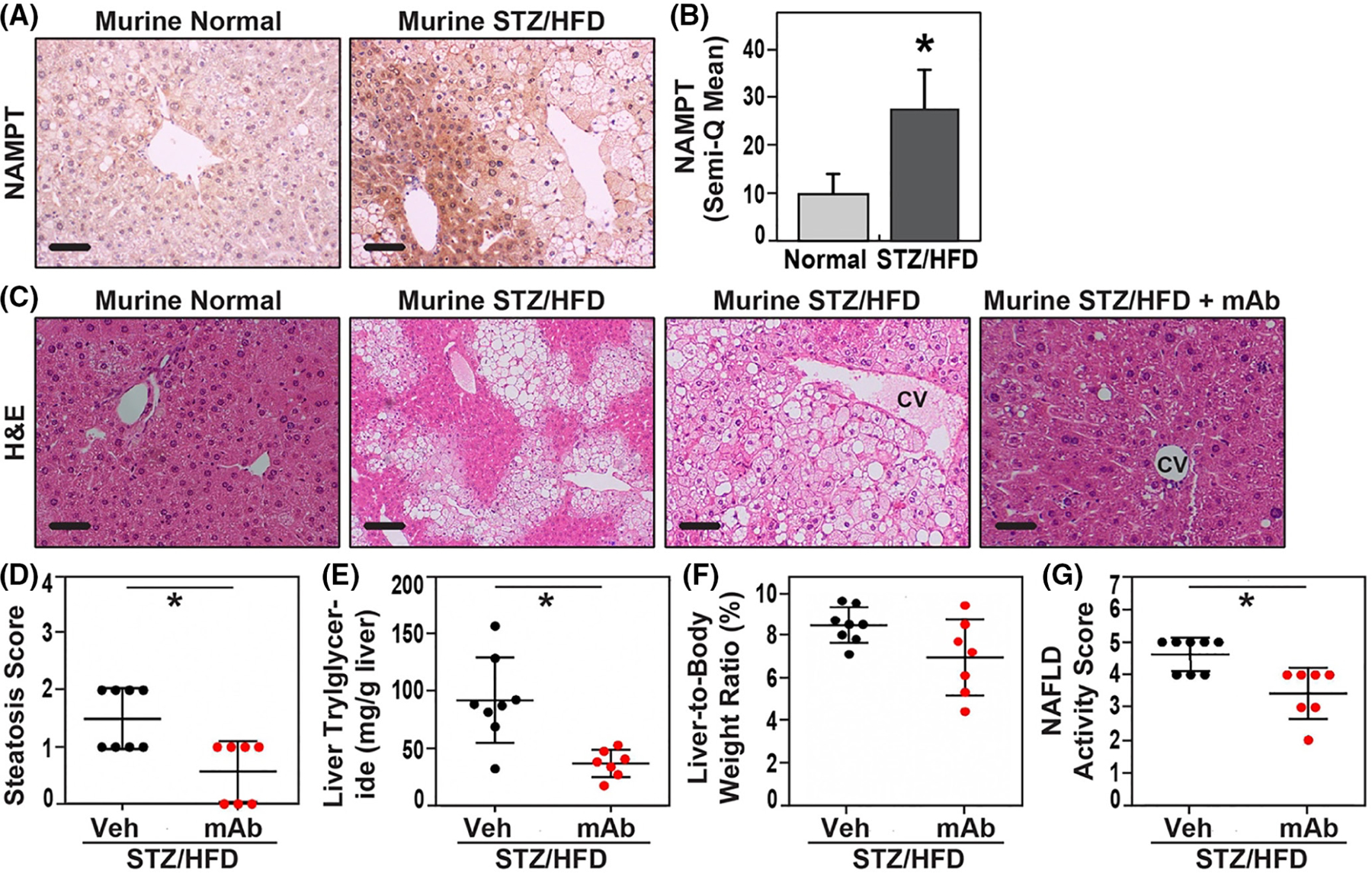
Role of eNAMPT in preclinical murine model of STZ/HFD-induced hepatic steatosis and NASH. (A) IHC for NAMPT showing low levels of NAMPT expression as mild staining in the control normal liver hepatocytes (Murine normal). NAMPT hepatic expression was markedly increased in NAFLD STZ/HFD livers showing strong NAMPT IHC staining in hepatocytes (Murine STZ/HFD). (B) Quantitation of NAMPT immunostaining intensity in hepatocytes showed significantly higher NAMPT expression levels in NAFLD STZ/HFD livers compared to normal control murine livers (*n* = 20, *p* < .05). (C) H&E staining showing normal liver in mice with control diet (Murine Normal). Control STZ/HFD mice developed pathological livers with severe pericentral/perivenous macrovesicular and microvesicular steatosis. STZ/HFD mice treated with IP anti-eNAMPT ALT-100 mAb, 0.4 mg/kg for 4 weeks, show marked reduction in hepatic steatosis. Control STZ/HFD mice were administered IP IgG with an identical dosing schedule. (D–G) Quantitative measures of the steatosis score (D), liver triglyceride level (E), liver-to-body weight ratio (F), and NAFLD activity score (NAS) (G) were significantly reduced in IP ALT-100 mAb-treated NAFLD mice compared to disease control NAFLD mice treated with IP IgG (*p* < .05, respectively) (bar—20 μm).

We next assessed NAMPT expression in the STZ/HFD NAFLD model which combines low-dose streptozotocin (STZ) with initiation of a high-fat diet beginning at 4 weeks. IHC for NAMPT expression in STZ/HFD-induced NAFLD livers showed significantly elevated intensity of NAMPT immunostaining compared to the mild NAMPT immunostaining in control livers (Figure 3A), findings verified by liver staining quantification (*n* = 16, *p* < .05) (Figure 3B). Examination of hepatic histology in STZ/HFD mice at 12 weeks revealed excess hepatic lipid accumulation, macrovesicular and microvesicular lipid vacuoles in hepatocytes, pericentral steatosis, hepatocyte ballooning injury, and lobular inflammation (Figure 3C, second and third panels) consistent with acute hepatitis and hepatocyte injury, histologic characteristics similar to human NAFLD histology. STZ/HFD mice also exhibited significantly increased steatosis scores (Figure 3D), elevated triglyceride levels in liver tissues (Figure 3E), and increased liver-to-body weight ratios (Figure 3F). Integration of these histologic data resulted in a markedly elevated NAFLD activity score (NAS of ~5) in STZ/HFD mice compared to control regular-diet-fed mice (NAS of up to 1) (Figure 3G). As NAS is a key clinical or preclinical endpoint for assessing the activity of NASH,^42^ these studies confirm that the murine NAFLD model histologically recapitulates human NAFLD.

**FIGURE 4:**
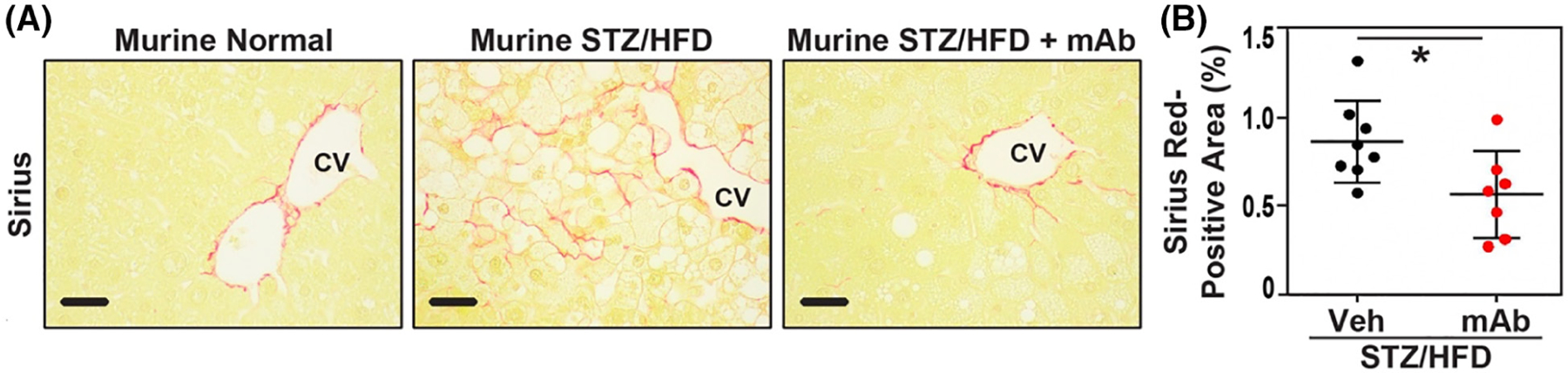
(A) STZ/HFD mice exhibit hepatic fibrosis which is reduced by the eNAMPT-neutralizing ALT-100 mAb. In normal liver (Murine normal), Sirius Red only stained the wall of central vein and portal tract without staining around hepatocytes. In NAFLD murine models (Murine STZ/HFD), fibrosis shown by Sirius Red stain progressed to pericentral perihepatocellular area as often observed in human NAFLD liver. Treatment with ALT-100 significantly prevented pericellular fibrosis (Murine STZ/HFD + mAb). (B) Quantitative measures of the severity of fibrosis by Sirius red staining positive area were significantly reduced in IP ALT-100 mAb-treated NAFLD mice compared to disease control NAFLD mice treated with IP IgG (*p* < .05) (bar—20 μm).

In addition to H&E staining, collagen deposition and fibrosis were determined using Sirius Red staining of STZ/HFD and control liver sections. Sirius Red staining in normal livers was confined to the walls of central veins and portal tracts but without peri hepatocyte staining (Figure 4A, first panel). In contrast, Sirius red staining of liver sections from STZ/HFD mice showed strong positivity in the pericentral region of the liver lobule with increasing pericellular hepatic fibrosis (Figure 4A, second panel).

**FIGURE 5:**
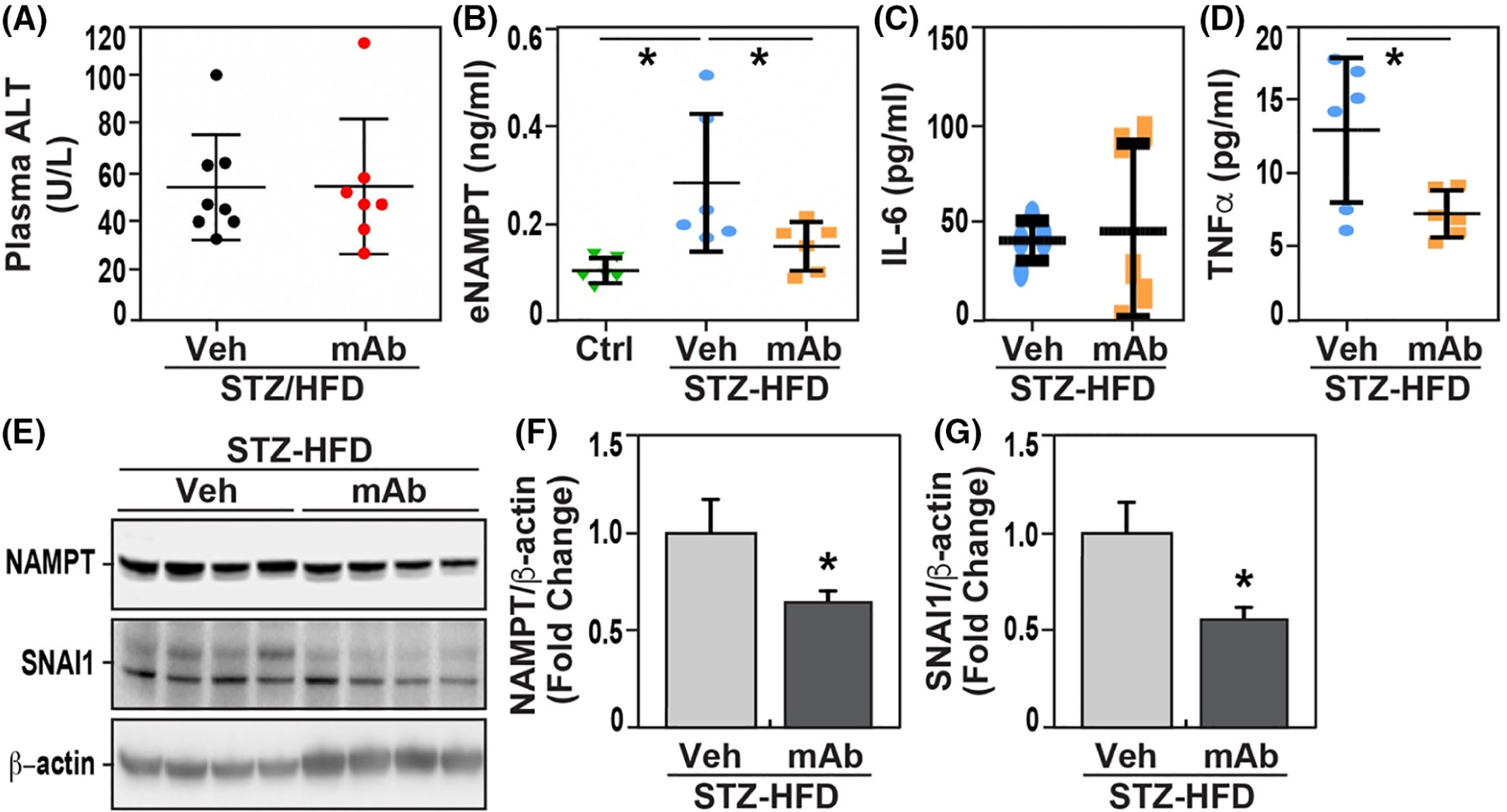
eNAMPT-neutralizing ALT-100 mAb reduces plasma cytokine levels and biochemical indices of hepatic fibrosis in STZ/HFD mice. (A) Plasma ALT levels were elevated in STZ/HFD compared to normal mice with levels similar in vehicle-treated NASH mice compared to ALT-100 mAb NASH mice. (B) Elevated plasma eNAMPT levels in STZ-HFD mice were significantly reduced in ALT-100 mAb-treated mice (*p < .05 vs. Ctrl, vs. Veh/STZ-HFD, respectively). (C) Plasma levels of IL-6 in STZ-HFD mice were similar in mice receiving the ALT-100 mAb. (D) Increased plasma levels of TNFα in STZ-HFD mice were significantly reduced in mice receiving the ALT-100 mAb (*p < .05 vs. Veh/STZ-HFD). (E–G) Quantitation of NAMPT immunoblot intensity in hepatic lysates from STZ-HFD mice showed that treatment with ALT-100 resulted in significantly decreased expression of NAMPT and SNAi1 (*p < .05 vs. Veh/STZ-HFD).

Finally, compared to normal mice, STZ/HFD-induced NAFLD mice demonstrated significantly increased serum levels of alanine transaminase (ALT, normal 20–45 U/L, Figure 5A) as well as plasma levels of eNAMPT (Figure 5B), IL-6 (Figure 5C), and TNFα (Figure 5D). The increased levels of NAMPT tissue staining (Figure 3A,B) and eNAMPT plasma levels (212 ± 25 pg/mL vs. 102 ± 0.4 pg/mL) (Figure 5B) were corroborated by Western blot analysis of liver lysates from control and STZ/HFD mice with increased NAMPT protein immunoreactivity in NAFLD hepatic tissues (Figure 5E,F).

### 3.3 | Effect of eNAMPT neutralization (ALT-100 mAb) on NASH severity and hepatic fibrosis in STZ/HFD mice

In preclinical models of inflammatory disease, we have previously demonstrated the critical role of eNAMPT-induced TLR4 signaling in determining the severity of several inflammatory disorders (ARDS, radiation lung injury, pulmonary hypertension, and lupus vasculitis), utilizing the humanized eNAMPT-neutralizing mAb, ALT-100.^14,18,21,24,25^ The therapeutic efficacy of ALT-100 mAb (0.4 mg/kg, once per week IP) was assessed in the STZ/HFD NAFLD model with ALT-100 mAb delivery beginning at week 9 and continued weekly through week 12. STZ/HFD NAFLD mice receiving ALT-100 mAb treatment demonstrated significant disappearance of hepatocyte lipid accumulation and intrahepatic fat vacuole steatosis and near absence of hepatocyte ballooning and lobular inflammation (Figure 3C, fourth panel). ALT-100 mAb-treated mice displayed significantly reduced hepatic parenchymal infiltration of immune cells including macrophages, neutrophils, and lymphocytes filtration compared to untreated mice (Figure 2S). These results culminated in the marked reduction of the NAFLD activity scores (NAS) (Figure 3G), liver triglycerides, and steatosis scores (Figure 3D,E) (Figure 1S).

The ALT-100 mAb also effectively reduced evidence of progression from steatosis to hepatic fibrosis as STZ/HFD mice receiving the eNAMPT mAb showed significantly reduced Sirius Red-positive areas of hepatic fibrosis, with pericentral/pericellular staining nearly absent (Figure 4A,B, third panel). Quantitation of Sirius Red staining intensity of ALT-100 mAb-treated mice showed a significant decrease in the Sirius-positive fibrosis area (0.56 ± 0.24%, *n* = 7) compared to the control mice (0.86 ± 0.23%, *n* = 8).

Examination of blood-derived indices of NASH severity in STZ/HFD mice revealed plasma ALT and IL-6 levels to be similar in vehicle-treated and ALT-100 mAb-treated NAFLD mice (Figure 5A,C). However, in contrast, the elevated plasma levels of both eNAMPT and TNFα in STZ/HFD mice were significantly attenuated in ALT-100 mAb-treated mice (Figure 5B,D). Biochemical examination of hepatic lysates from STZ-HFD mice receiving treatment with ALT-100 mAb showed significant decreases in hepatic expression of NAMPT and SNAI1, a key factor in epithelial/endothelial mesenchymal transition and progression to tissue fibrosis^43^ (Figure 5E–G).

## 4 | DISCUSSION

Accumulating preclinical evidence implicates the novel DAMP, eNAMPT, as an essential participant in multiorgan inflammatory injury and fibrosis^13,18,21,24,25,44–46^ and cancer.^12,28,29,32^ Although the influence of eNAMPT on NAFLD progression to NASH/hepatic fibrosis has not been previously studied, TLR4, the PRR and eNAMPT receptor, is involved in dysregulated inflammatory and metabolic lipid pathways that alter the onset of NAFLD,^47,48^ chronic liver injury, and fibrosis.^49^ Reduced TLR4 expression enhances fatty acid oxidation in the liver and prevents triglyceride accumulation^47^ and DAMPs from the portal circulation including TLR4 agonists (HMGB1, defensins, heat shock protein, and hyaluronic acid) augmented hepatic steatosis and inflammation.^50,51^ Previous studies in vitro and in vivo well characterized the roles of eNAMPT/TLR4/NFkb signaling under physiological and pathobiological conditions.^12,52–54^ The current study leveraged the availability of an eNAMPT-neutralizing humanized mAb, ALT-100, a valuable tool utilized in preclinical models of prostate cancer,^28,29^ pulmonary hypertension,^24^ radiation pneumonitis/fibrosis,^13,21^ and acute lung injury,^18^ to assess the utility of eNAMPT/TLR4 targeting as a therapeutic approach to retard NAFLD progression to hepatic fibrosis. In a 12-week murine STZ/HFD model of NAFLD, STZ/HFD NAFLD mice receiving the eNAMPT-neutralizing ALT-100 mAb from weeks 9–12 exhibited significantly reduced NASH severity compared to untreated mice. This was captured by reductions in the NAFLD activity score (NAS), reductions in multiple confirmatory indices of liver steatosis, and clear attenuation of NASH progression to hepatic fibrosis. These studies strongly suggest the eNAMPT/TLR4 inflammatory cascade as a major contributor to NAFLD progression and hepatic fibrosis.

The present study demonstrates that human subjects with clinically and pathologically diagnosed NAFLD exhibit significantly increased hepatic NAMPT expression and plasma eNAMPT levels. Secreted eNAMPT is elevated in humans with obesity,^55^ type 2 DM,^56^ and metabolic syndrome and correlates with insulin resistance, glucose metabolism,^31^ and cardiovascular diseases.^56^ In high-fat diet-fed mice, anti-eNAMPT antibodies prevented the development of type 2 DM and reduced glucose intolerance and insulin resistance.^57,58^ In addition, intravenously injected eNAMPT directly contributed to hepatic inflammatory cell infiltration, cytokine production, and fibrosis in a methionine-deficient NASH model.^59^

Innovative therapeutic advances in NAFLD management, including NASH therapies in phase III trials,^11,60^ highlight the role of inflammatory pathway activation^61^ and oxidative/proteotoxic stress^6,62^ in hepatic fibrogenesis and progression to hepatocellular carcinoma. Our data provide critical new evidence of involvement of the eNAMPT/TLR4 inflammatory cascade in NAFLD and strongly indicate the eNAMPT-neutralizing ALT-100 mAb therapy as a novel effective therapy in NAFLD to prevent progression to NASH and fibrosis. Whereas eNAMPT has not been previously targeted in human studies, intracellular NAMPT (iNAMPT), the essential rate-limiting enzyme in nicotinamide adenine dinucleotide (NAD) biosynthesis salvage pathway, has been the focus of numerous cancer therapeutic trials using iNAMPT enzymatic inhibitors (GMX-1776/CHS-828, APO-866/Daporinad/FK866) which were uniformly unsuccessful due to limited therapeutic benefit and significant dose-limiting toxicities.^63,64^ These findings highlight the essential role of iNAMPT in normal human cell survival under hypoxia or nutritional depletion.^31^ In mouse models of NAFLD, iNAMPT ameliorates the development of NAFLD by generating NAD+, increasing SIRT1 pathway activity, and protecting hepatocytes from apoptosis.^65–67^ As the ALT-100 mAb, delivered IV or subcutaneously, targets circulating extracellular eNAMPT, an impact on intracellular NAD metabolism is unlikely. This is supported by Investigational New Drug (IND)-enabling pharmacokinetic studies in rats and minipigs utilizing the humanized GMP-manufactured ALT-100 mAb which demonstrated the complete absence of toxicity and a therapeutic half-life of ALT-100 mAb of 21–30 days. The feasibility of targeting eNAMPT/TLR4 inflammatory cascade as a therapeutic strategy for NAFLD will be underscored by ongoing Phase 1A safety and pharmacokinetic studies in healthy human volunteers.

There are several limitations to our study. First, our findings are limited to the HFD/STZ model of NAFLD. Future studies will attempt to further verify the protective role of ALT-100 mAb in NASH progression by using the AMLN^68^ and CDA-HFD-induced NASH mice model.^69^ Second, we only studied a select number of inflammation- and fibrosis-related genes/proteins which proved to be significantly dysregulated in IL-6, Ang-2, IL-1RA, TNFα, and SNAI1 in plasma/liver tissues. Future studies will endeavor to utilize genome-wide profiling to expand upon the effects of the ALT-100 mAb on the location, expression, and transcriptional regulations of these and additional hepatic-related related genes. Third, oxidative and ER stresses are well-known and key features of NASH but were not examined directly in the present study but will certainly be a targeted pathway in future studies utilizing ALT-100 mAb efficacy in NASH. Finally, although the contribution of TLR4/NFkB signaling to NASH and hepatic fibrosis is well known, in future studies, we will validate the protective role of ALT-100 mAb via eNAMPT/TLR4 utilizing TLR4 knockdown or inhibitors.

In summary, our study supports eNAMPT/TLR4 signaling as a clinically relevant, highly druggable therapeutic target with an eNAMPT-neutralizing biologic therapy identified as a potential strategy to retard the progression of NAFLD from steatosis to fibrosis. We speculate that eNAMPT is generated and released from leukocytes, endothelial cells, and enterocytes in the “leaky gut” as well as hepatocytes in the NAFLD liver or mesenteric adipose tissues. Local and circulating eNAMPT directly activates the TLR4/NFκB-dependent inflammatory cascade to contribute to hepatic transition from steatosis to NASH and fibrosis. While the study of ALT-100 mAb on the development of hepatocellular carcinoma was beyond our scope, we hypothesize that eNAMPT inhibition will be protective against HCC development. Clearly, additional preclinical/clinical studies are required to fully define the efficacy of eNAMPT-neutralizing therapies in halting NAFLD progression and long-term manifestations of advanced NAFLD such as cirrhosis and HCC.

## Supplementary Material

Supplemental

## Figures and Tables

**TABLE 1:** Clinical characteristics of NAFLD subjects examined for tissue NAMPT expression.

Case	Fibrosis stage	NAS (1–8)	Age	Gender	BMI	Diabetes	Ethnicity
1	Stage 0	3	17	M	38.5	No	Caucasian
2	Stage 1a	4	32	F	35.1	No	Hispanic
3	Stage 2	6	55	M	30.2	Type 2	Hispanic
4	Stage 3	5	46	M	33.4	Type 2	Caucasian
5	Stage 4	7	61	F	28.6	Type 2	Caucasian

**TABLE 2:** Clinical characteristics of NAFLD and healthy subjects assessed for plasma biomarker measurements.

Characteristics	NAFLD (*N* = 67)	Healthy controls (*N* = 78)
Sex (male, female)	22, 45	47, 32
Age (years), mean ± SD	64.7 ± 8.3	53.7 ± 16.8
African American (*n*, %)	1 (1.5%)	8 (10.3)
European American (*n*, %)	56 (82.1%)	45 (57.7)
Native American (*n*, %)	6 (8.9)	1 (1.2)
Ethnicity: Latino (*n*, %)	27 (41.8)	10 (12.8)
Type 2 diabetes (*n*, %)	35 (52.2)	NA
Hypertension (*n*, %)	47 (70.1)	NA
Dyslipidemia	27 (40.3)	NA

## Data Availability

The data that support the findings of this study are available from the corresponding author upon reasonable request.

## Abbreviations:

ARDS: acute respiratory distress syndrome
eNAMPT: extracellular nicotinamide phosphoribosyltransferase
DAMP: damage-associated molecular pattern protein
HCC: hepatocellular carcinoma
IL-1RA: interleukin 1 receptor antagonist
mAb: monoclonal antibody
NAD: nicotinamide adenine dinucleotide
NAFLD: nonalcoholic fatty liver disease
NAS: NAFLD activity score
NASH: nonalcoholic steatohepatitis
PRR: pattern recognition receptor
STZ/HFD: streptozotocin/high-fat diet
TLR4: toll-like receptor 4
