# Supplementary figures and images for: Involvement of eNAMPT/TLR4 inflammatory signaling in progression of non-alcoholic fatty liver disease, steatohepatitis, and fibrosis
Source: FASEB J. Author manuscript; Available in PMC 2024 Jul 23. (PMC11265521; doi:10.1096/fj.202201972RR)

Supplemental Figure 1:

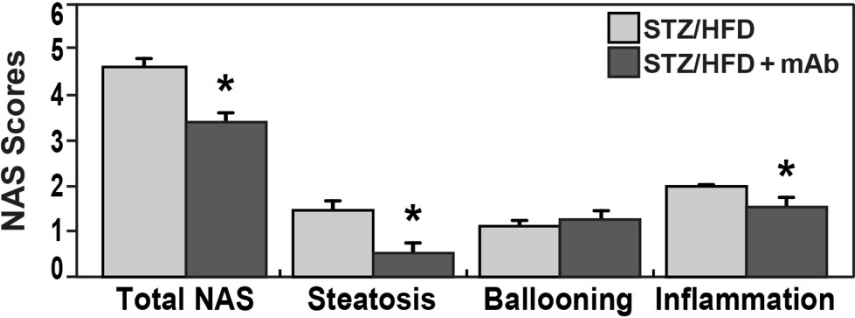

**Supplemental Figure 2:**

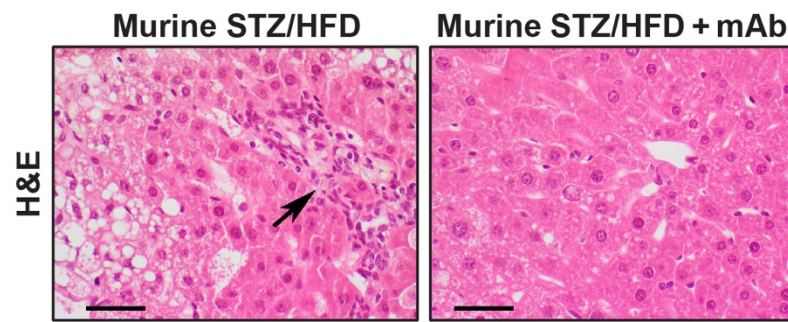

Supplement: Supplemental [file NIHMS2005350-supplement-Supplemental.pdf]
